# Comparative studies on genital infections and antimicrobial susceptibility patterns of isolates from camels (*Camelus dromedarius*) and cows (*Bos indicus*) in Maiduguri, north-eastern Nigeria

**DOI:** 10.1186/2193-1801-3-91

**Published:** 2014-02-15

**Authors:** Gideon Dauda Mshelia, Godfrey Okpaje, Yepmo Andre Casimir Voltaire, Godwin Onyeamaechi Egwu

**Affiliations:** Department of Veterinary Surgery and Theriogenology, Faculty of Veterinary Medicine, University of Maiduguri, PMB 1069 Maiduguri, Nigeria; Department of Veterinary Medicine, Faculty of Veterinary Medicine, University of Maiduguri, PMB 1069 Maiduguri, Nigeria

**Keywords:** Antimicrobial susceptibility, Bacteria, Camels, Cows, Genitalia, Nigeria

## Abstract

A total of 160 genitalia of Camels and cows were investigated in Maiduguri, north-eastern Nigeria to compare bacterial isolates and the antibacterial susceptibilities of some of the isolates. *Streptococcus* (*Str*.) *pyogenes* (31%), *Escherichia* (*E*.) *coli* (24%) and *Staphylococcus* (*S*.) *aureus* (20%) were the most common vaginal bacterial isolates in camels; while *E. coli* (*73*%), *Str. pyogenes* (*18*%) and *S. aureus* (*11*%) were the most frequent isolates in the cows. Of the 78 uterine isolates recovered in this study, *E. coli* was the most prominent in camels (8%) and cows (54%). The overall weight of genital infection in all camels and cows examined was highest (P < 0.05) with *E. coli* (79%), but there was no difference (P > 0.05) between vaginal and uterine bacterial isolates from camels and cows in this study. The Relative Risk (RR) for an infection of the vagina with *E coli* (3.04, 95% Confidence Interval (CI): 2.104 to 4.398, P < 0.0001) is more in cows compared to the camel, but the RR for vaginal infection with *S. aureus* and *Str. pyogenes* were lower in cows compared to Camels. The *E. coli* and *S. aureus* isolates were highly susceptible to the antimicrobial agents tested. Therefore effective management of reproductive disorders associated with these pathogens can be achieved with proper use of these antimicrobial agents in these animal species

## Introduction

The camelidae family has been described previously (Mouchira 2009). The camelidae are resilient to adverse weather conditions and are able to stay long without water and food, which have made them to become more important as a source of meat and draught power in sub-Saharan Africa (Mouchira 2009). Camel meat is daily being accepted in northern Nigeria and they may likely replace cattle as the main animal protein source for human population in Nigeria (Srikandakumar et al. 2001).

At the moment these two livestock resources provide the meat consumed in northern Nigeria, so there is need to continue to intensify their production to meet up with the ever increasing demand for this product in the country. These animals are bred majorly using natural mating which is characterized by low reproductive performance, mostly associated with puerperal infections of the genital tracts (Sheldon et al. 2006). Therefore, it is important to routinely assess the genital bacterial status of breeding animals as part of custom reproductive improvement programme.

The bacteria colonizing the genital tract of the female camel (*Camelus dromedarius*) have been shown to be the major causes of reproductive disorders in this species (Ali et al. 2010; Tibary et al. 2006; Wernery and Kumar 1994). Evidence for the presence of a variety of uterine bacterial isolates has been demonstrated from studies using slaughterhouse materials. Some were isolated from the uterus of barren camels, but their responsibility as primary causes of uterine infections has been doubted (Enany et al. 1990; Hussein et al. 2006). Postpartum infections in cattle are eliminated within 2–4 weeks of parturition (Hussain et al. 1990). However, some uterine pathogens persist to cause subclinical endometritis in this species (Fourichon et al. 2000; Heuwieser et al. 2000).

The disruption of the immune status during the periparturient period in cattle renders the uterus vulnerable to ascending infections with opportunistic bacteria from the vagina and the animals’ environment. These infections rise during this period compared with other stages of the reproductive cycle (Sheldon et al. 2002; Singh et al. 2008) requiring antimicrobial treatment for clearance (Drillich 2006; Sheldon et al. 2009) and improvement in subsequent fertility in these species. However, the inappropriate use of antimicrobial agents for the treatment of infective reproductive disorders in camels and cows led to increased bacterial contaminations of the genital environment (Tibary and Anouassi 2001; Potter et al. 2010; Gani et al. 2008). The pattern in the pastoral husbandry system is changing particularly in the semi-arid regions in Nigeria (Blench 1999), associated with the scarcity of food resources and drinking water at certain times of the year. So, camels could often be seen being herded together with cattle, donkeys and other small ruminant species at some watering points and market places (El-Yuguda et al. 2010; Markemann and Zarate 2010), which facilitates the transmission of infections between these species. Unlike cattle, there is dearth of information regarding infective pathogens colonizing the genital tracts in camels. This study was therefore designed to identify and compare the bacterial species colonizing the vagina and uterus of camels and cows in north-eastern Nigeria; and to determine their antimicrobial susceptibilities for effective management of reproductive disorders in these species.

## Materials and methods

### Animals and samples collection

The study was conducted in Maiduguri, Borno State in north eastern Nigeria. The parity and other reproductive histories of the she-camels (*Camelus dromedarius*) and cows (*Bos indicus*) were unknown, but they were culled from pastoral herds using natural breeding. The size of the samples collected was determined according to the formula provided by Thrustfield ( 2005):

where n = required sample size, Pexp = expected prevalence and d = desired precision. The calculation was based on 95% level of confidence (CL), 5% margin of error, and with the assumption that 50% of the genitalia will be infected with bacteria, Accordingly, a total of 160 (80 each from camels and cows) were collected in Maiduguri municipal abattoir. The genital tracts were collected twice weekly and transferred on ice in clean polyethelyne bags to the diagnostic laboratory within two hrs of collection. This study was approved and carried out in accordance with the ethical provisions of the faculty of Veterinary Medicine, University of Maiduguri.

### Bacterial culture and isolation

Bacteriological examination was carried out on the vagina and uterus using standard protocols (Cheesbrough 2000). Swab samples were collected routinely from the vagina (Amin et al. 1996) and uterus (Azawi et al. 2010). All the samples were inoculated onto blood and MacConkey’s agar plates, and incubated at 37°C for 24–48 hrs. Suspect colonies were examined for colony morphology, Grams characteristics and motility. Gram negative bacilli and Gram positive cocci were further subjected to catalase, oxidase and coagulase tests as well as standard biochemical tests (Cowan and Steel 1993; Koneman et al. 2005) to identify the isolates.

### Antimicrobial susceptibility test

The disk diffusion test was used to determine the antimicrobial susceptibility of the confirmed bacterial isolates against panels of antimicrobial agents. This test was performed on the *E. coli* and *S. aureus* isolates recovered in the present study. The antimicrobial agents tested were Amoxycillin (25 μg), Ampicillin (10 μg), Amoxycillin-Clavulanate (30 μg), Cephalexin (30 μg), Ciprofloxacin (10 μg), Clindamycin (10 μg) and Co-trimoxazole (25 μg). Others include Erythromycin (5 μg), Gentamycin (30 μg), Nalidixic acid (30 μg), Norfloxacin (10 μg), Ofloxacin (5 μg), Pefloxacin (5 μg) and Streptomycin (10 μg). The antimicrogram pattern was determined according to the Kirby Bauer procedure described by Demissie ( 2011). Briefly, pure colonies of bacterial growth were suspended in tubes containing 5mls of Brain Heart infusion broth (Sigma-Aldrich, UK) and adjusted to 0.5 McFarland turbidity standards. 10 μl of the diluted bacterial suspensions were transferred to Mueller Hinton agar plates (BBL®, Becton Dickinson, USA) using sterile cotton swab applicator sticks. Excess fluid was squeezed out by rotating the swabs against the sides of the tubes. The plates were then inoculated uniformly by rubbing the swabs against the entire agar surfaces and allowed to dry. The impregnated antimicrobial discs (Optun Laboratories Nig Ltd., Lagos, Nigeria) were applied to the surfaces of the inoculated plates using sterile forceps. All the discs were gently pressed with forceps to ensure complete contact with the agar surface. The discs were placed 1.5 cm away from the edges of the plates and 3 cm away from each other with the guide of a template placed under the petri-dish. The plates were then inverted and incubated aerobically for 24 hr at 37°C. The zones of inhibition of bacteria by the antimicrobial discs were measured in millimeters using a caliper on the underside of the plates. The susceptibility of the bacteria was determined based on the breakpoints recommended by the Clinical Laboratory Standards Institute (CLSI 2011).

### Data analysis

The data on the genital bacteria and their isolation rates were analysed using descriptive statistics. The Chi-square test was used to test the differences in the percentages of the isolates, while the relative risks (RR) for an infection with bacteria were analysed and the significance tested with the Fisher’s Exact Test using Graph Pad Prism Statistical Software version 5.04 (GraphPad Software 2010). Unless otherwise stated P value was considered significant at <0.05.

## Results

### Vaginal and uterine bacterial isolates

The bacteria colonizing the vagina and uterus in camels and cows are shown in Table 1. Out of a total of 75 bacterial isolates from camels (n = 80), 66 of those were recovered from the vagina and made up of 25 (31.3%) *Str. pyogenes*, 19 (23.8%) *E. coli*, 16 (20%) *S. aureus*, 5 (6.3%) *Proteus* spp and 1 (1.3%) *Corynebacterium* spp. Nine of the isolates were recovered from the uterus. Out of these number, 6 (7.5%) were *E. coli*, 1 (1.25%) *S. aureus*, 1 (1.25%) *Str. pyogenes* and 1 (1.25%) *Corynebacterium* spp*.* There were four gravid camelidae uteri examined from this specie, but no bacteria were isolated from them.

**Table 1:** **Vaginal and uterine bacterial isolates from Cows and Camels slaughtered in north-eastern Nigeria**

Isolates	Cows (n = 80)		Camels(n = 80)		RR
	n	%	n	%	
VAGINA					
*Streptococcus pyogenes*	14	18	25	31	(0.58, 95% CI: 0.3485 to 0.9674, P = 0.0479),
*Staphylococcus aureus*	9	11	16	20	(0.55, 95% CI: 0.2782 to 1.087, P = 0.1170)
*Staphylococcus spp	19	23	NI		
*Escherichia coli*	58	73	19	24	(3.04**, 95% CI: 2.104 to 4.398, P < 0.0001)
*Proteus* spp	11	14	5	6	
*Corynebacterium* spp	NI		1	1	
UTERUS					
*Streptococcus pyogenes*	11	14	1	1	(14.0**, 95% CI: 1.875 to 104.51, P = 0.0006)
*Staphylococcus aureus*	9	11	1	1	(11.0**, 95% CI: 1.446 to 83.652; P = 0.0050)
*Staphylococcus spp	6	8	NI		
*Escherichia coli*	43	54	6	8	(6.750**, 95% CI: 3.389 to 13.444, P < 0.0001)
*Proteus* spp	3	4	NI		
*Corynebacterium* spp	NI		1	1	

*Abbreviations*: NI, not isolated, *other Staphylococcus species, RR, relative risk for an infection with bacteria in cows compared to camels, CI, Confidence Interval, **within a column, values are statistically significant.

In the cows (n = 80), there were 111 isolates recovered from the vagina which include 14 (17.5%) *Str. pyogenes*, 9 (11.3%) *S. aureus*, 58 (72.5%) *E. coli*, 11 (13.8%) *Proteus* spp and 19 (23.3%) other *Staphylococcal* spp*.* The uterine cultures yielded 69 bacterial isolates, 43 (53.8%) of which were *E. coli*, 11 (13.8%) *Str. pyogenes*, 9 (*11.3*%) *S. aureus*, 3 (3.8%) *Proteus* spp and 6 (*7.5*%) other *Staphylococcal* spp. Two gravid uteri were examined from this specie, from which, *E coli* and *Staphylococcal* spp were isolated from 2/2 and 1/2 of the uteri. The overall weight of genital infection was calculated; and the highest infection rate was associated with *E. coli* (79%) followed by *Str. pyogenes* (32%) and *S. aureus* (25%) in all the animals investigated in the present study (Table 2).

**Table 2:** **The overall weight of genital infection with bacteria isolated from Cows and Camels slaughtered in north-eastern Nigeria**

Isolates	***E. coli***	***Str. pyogenes***	***S. aureus***
COWS (n = 80)	101	25	23
CAMELS (n = 80)	25	26	17
Total isolates (Infection rate)	126 (79%)	51 (32%)	40 (25%)

Abbreviation: n, number of animals investigated.

### Relative risk (RR) analysis

The risks of infection of the vagina and uterus with bacteria were calculated for the cow and camels. The RR for an infection of the vagina with *E. coli* (3.04, 95% CI: 2.104 to 4.398, P < 0.0001) is higher in cows compared to camels, while the RR for *S. aureus* (0.55, 95% CI: 0.2782 to 1.087, P = 0.1170) and *Str. pyogenes* (0.58, 95% CI: 0.3485 to 0.9674, P = 0.0479) were lower in cows compared to camels. However, the RR for uterine infection with *Str. pyogenes* (14.0, 95% CI: 1.875 to 104.51, P = 0.0006) is higher in cows compared to camels, so also with *E. coli* and *S. aureus* (Table 1).

### Antimicrobial susceptibility test

**Figure 1:**
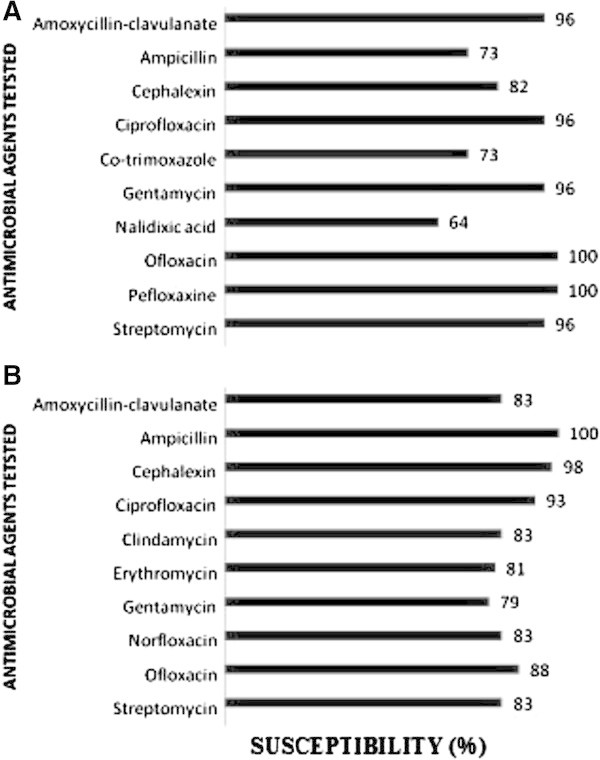
**Antimicrobial susceptibility patterns for bacterial isolates. (A)** Shows the susceptibility of *E. coli,* and **(B)** the susceptibility of *S. aureus*.

The antimicrobial susceptibility of *E. coli* was highest (100%) against Pefloxacine and Ofloxacin, and 96% against Amoxycillin-clavulanate, Ciprofloxacin, Gentamycin and Streptomycin. This pathogen was susceptible to the other antimicrobial drugs at rates ranging from 73–82%. The lowest susceptibility was observed against Nalidixic acid (64%; Figure 1A). *S. aureus* had the highest susceptibility against Ampicillin 100%, followed by Cephalexin (98%) and Ciprofloxacin (93%) and Ofloxacin (88%). The susceptibility of *S. aureus* against most of the other drugs ranged from 81–83%, with the lowest susceptibility recorded against Gentamycin (79%; Figure 1B).

## Discussion

The information regarding bacteria causing genital infections in female dromedary camels is scarce (Ali et al. 2010). However, evidence implicating bacterial infections as causes of endometritis has been reported, and a variety of these bacterial species have been recovered from the uteri of infertile camelids (Tibary et al. 2006; Wernery and Kumar 1994). Out of the vaginal bacterial isolates recovered from camels in this study, *Str. pyogenes, S. aureus* and *E. coli* were the most common. The isolation rate of *Str. pyogenes* (31%) alone was higher (P < 0.0%) compared to the rates for *E. coli* and *S. aureus*. This bacteria has been isolated previously in camels (Tibary and Anouassi 2001) suffering from endometritis (Wernery and Kumar 1994) and in those with mucopurulent or purulent vaginal discharges (Ali et al. 2010). *Proteus* spp and *Corynebacterium* spp were also isolated, with the later having the lowest isolation rate (1%).

In cows, *E. coli* was the most commonly isolated bacteria from the vagina. Other isolates including Staphylococcal organisms, *Str. pyogenes* and *Proteus* spp were moderately isolated from this species, but *Corynebacterium* spp was not isolated. The rate of isolation of *E. coli* observed in the present study (73%) was highly significant, and accounted for more than all the other bacterial isolates put together. Previously, *E. coli* was thought to be a non-specific pathogen associated with endometritis in mares and cows (Arthur et al. 2000), but they were recently isolated in camels with purulent discharges (Ali et al. 2010). The presence of this bacterium at the present level of infection in the vagina could likely lead to metritis as a consequence of ascending uterine infection during breeding. Specific strains of *E. coli* have recently been shown to be pathogenic for the endometrium, causing Pelvic Inflammatory Disease (PID) in cattle (Sheldon et al. 2010). But the role of this bacterium in the pathogenesis of metritis especially in camels merits further studies.

The uterine bacterial isolates observed in camels in the present study are similar to those observed in the vagina, and they concurred with the findings of Yagoub ( 2005) who reported *S. aureus, E. coli, Klebsiella* sp*, Proteus* sp*, Corynebacterium* sp and *Streptococcus* sp as the main bacterial isolates from several cases of uterine infections in camels. The most common isolate in the present study was *E. coli* with an isolation rate of 6%. The isolation rates of the uterine bacteria observed in the present study were lower compared to those from the vagina, and more so in the camels than the cows. Unlike the cows, *Proteus* sp was not isolated in the camelids uterus, but *Str. pyogenes*, *S. aureus* and *Corynebacterium* sp were isolated at a minimal rate of 1% from all the camels examined. The result from the cows also showed that *E. coli* was the major bacteria isolated from the uterus. The isolation rate of this bacterium (54%) accounted for more than all the rates for the other bacteria isolated from the bovine uterus. Although uterine infection with *E. coli* is significant in the present study, the rate is lower in the uterus compared to the vagina, which is likely due to the continuous clearance of bacteria from the uterine lumen (Singh et al. 2008). Despite this low isolation rate, it is important to note that the isolation of pathogenic bacteria such as *E. coli* in the present study portends a risk factor for lowered reproductive efficiency because of increased inflammatory reactions and possible damages to the uterine tissues (Sheldon and Dobson 2004) by direct action of the bacteria or its toxins.

The data in the present study has shown that there was no difference (p > 0.05) between the bacterial species colonizing the vagina and uterus in camels and cows. But on the overall, the weight of infection with *E coli* (79%) in all the animals examined was more than the rates for *Str. pyogenes* and *S. aureus* put together.

Generally the pre-breeding and peri-parturient periods are known as the most critical for bacterial infection of the genital tract. This is due to the hormonal changes that make the uterus susceptible for ascending infections with resident bacteria colonizing the vagina (Singh et al. 2008). During these periods, the vagina is constantly being contaminated with bacteria from the environment and from faecal droppings that smear the vagina during breeding seasons. These and other contaminants from the male genitalia are introduced into the female vagina by stud bulls which can lead to uterine infections in vulnerable animals (Singh et al. 2008; Tibary and Anouassi 2001). Also, during the immediate period post partum the cervix is dilated (Sheldon and Dobson 2004) which allows bacteria to ascend from the vagina into the uterus, causing infections in 90% of cows by 21 days post partum (Sheldon et al. 2006). This could possibly explain the similarity between the types of vaginal and uterine bacteria isolated in the present study.

The occurrence of ovulation prior to the expulsion of exudates and debris from the uterus has been shown to favor heavy growth of bacteria in the uterine environment which leads to the retention of the corpus luteum (CL) and consequent impairment of the ability of the uterus to secret PGF2α (Kaneko et al. 2013). Although, there is continuous bacterial clearance and recontamination of the uterine lumen for up to 7 weeks postpartum (Singh et al. 2008), some bacteria still persist in the uterus triggering inflammatory responses and pathological changes. This delays uterine involution (Williams et al. 2005) thereby lowering fertility. The types of bacteria colonizing the uterus are likely to influence the severity of this condition (Singh et al. 2008) in countries where natural breeding is common practice.

The result of the present study also showed that *E. coli* and *S. aureus* were isolated from pregnant bovine uteri. This finding is interesting considering that the uterus is thought to be sterile during pregnancy (Singh et al. 2008), when the cervix is closed. Infections of the uterus carrying life pregnancies are the common causes of repeat breeding occasioned by conception failures (Ferreira et al. 2008; Gani et al. 2008). Furthermore, the isolation of *E. coli* from a gravid uterus is particularly important in this study because the bacterium is most frequently associated with uterine disease in cattle (Sheldon et al. 2002; Williams et al. 2005). Infections in the pregnant female could lead to abortion, prenatal-neonatal loss and stillbirth (Tibary et al. 2006). The persistence of such infections post partum is likely to contribute to the early demise of the CL with decrease in secretion of progesterone (P4) in the affected animal (Williams et al. 2007) which could also lead to pregnancy failures.

The number of bacteria colonizing the uterus and the level of uterine immune response are important determinants of uterine infections (Azawi 2008; Singh et al. 2008). When the immune status is lowered, the pathogenic bacteria adhere to the endometrial mucosa, get internalized and penetrate the epithelium. Alternatively, the bacteria can also release toxins that cause uterine diseases (Azawi 2008). The findings in the present study have shown that the risk of an infection of the vagina with *E. coli* is higher in the cows compared to the camels, but inversely so with *S. aureus* and *Str. pyogenes.*

Antimicrobial agents are commonly used in the management of reproductive failures in livestock (Drillich 2006). In Nigeria, they are used concurrently with prophylactic de-worming regimen in beef bull fattening schemes, but there is no special attention given to antibiosis with replacement heifers or breeding cows. The findings in the present study showed that *S. aureus* was highly susceptible to most of the antimicrobial agents tested, among which Ampicillin, Cephalexin, Ciprofloxacin and Ofloxacin were the most effective. This finding concurs with the observations made by Gani et al. ( 2008) who found Ciprofloxacin as one of the most effective antimicrobial agent against Staphyloccocal uterine infections in dairy cows. In one study, Fazlani et al. ( 2011) showed that *S. aureus* isolates from camels milk was moderately susceptible to amoxicillin, ampicillin, cephalexin and ofloxacin within the range of 64-86%. Serin et al. ( 2010) also demonstrated that *S. aureus* isolate from wrestling dromedary bulls was 100% susceptible to ciprofloxacin.

For *E. coli*, the susceptibility pattern to most of the antimicrobial agents tested was similar to what was observed with the *S. aureus* isolates, but the susceptibility was higher compared to those observed with the *S. aureus* isolates. The most effective antimicrobial agents observed include Ofloxacin and Pefloxacin, also Amoxycillin-clavulanate, Ciprofloxacin, Gentamycin and Streptomycin. This finding is similar to previous reports with isolates associated with genital infections in cattle and sheep (Gani et al. 2008; Goncuoglu et al. 2010), and from mastitic milk samples of camels (Fazlani et al. 2011).

Amongst the factors that have been reported to be contributing to uterine infections in camelids are overbreeding (excessive matings during the period of receptivity), postpartum complications and unhygienic gynaecological examination and manipulation (Tibary and Anouassi 2001). It has been highlighted that antimicrobial treatments have some beneficial effects on reproductive performance in livestock (Drillich et al. 2003). Therefore, at the current level of susceptibility of these bacteria to the antimicrobial agents tested in the present study, effective treatment could be achieved if these antimicrobial agents are used appropriately.

## Conclusions

The bacteria colonizing the genital tract are similar in camels and cows reared in north-eastern Nigeria. *E. coli* and *S. aureus* were amongst the most prevalent bacteria isolated, and they were found to be susceptible to the antimicrobial agents tested. It is advised that effective gynaecological evaluations should precede the initiation of antimicrobial treatments in order to minimize the development of antimicrobial resistant pathogenic strains in these species.
